# Codon Usage Provide Insights into the Adaptation of Rice Genes under Stress Condition

**DOI:** 10.3390/ijms24021098

**Published:** 2023-01-06

**Authors:** Swati Tyagi, Pramod Gorakhanath Kabade, Niranjani Gnanapragasam, Uma Maheshwar Singh, Anoop Kishor Singh Gurjar, Ashutosh Rai, Pallavi Sinha, Arvind Kumar, Vikas Kumar Singh

**Affiliations:** 1 International Rice Research Institute-South Asia Regional Centre (ISARC), Varanasi 221106, India; 2 International Rice Research Institute (IRRI)-South-Asia Hub, International Crops Research Institute for the Semi-Arid Tropics, Hyderabad 502324, India

**Keywords:** codon usage bias, abiotic stress, gene expression, mutational pressure, natural selection

## Abstract

Plants experience different stresses, i.e., abiotic, or biotic, and to combat them, plants re-program the expression of growth-, metabolism-, and resistance-related genes. These genes differ in their synonymous codon usage frequency and show codon usage bias. Here, we investigated the correlation among codon usage bias, gene expression, and underlying mechanisms in rice under abiotic and biotic stress conditions. The results indicated that genes with higher expression (up- or downregulated) levels had high GC content (≥60%), a low effective number of codon usage (≤40), and exhibited strong biases towards the codons with C/G at the third nucleotide position, irrespective of stress received. TTC, ATC, and CTC were the most preferred codons, while TAC, CAC, AAC, GAC, and TGC were moderately preferred under any stress (abiotic or biotic) condition. Additionally, downregulated genes are under mutational pressure (R^2^ ≥ 0.5) while upregulated genes are under natural selection pressure (R^2^ ≤ 0.5). Based on these results, we also identified the possible target codons that can be used to design an optimized set of genes with specific codons to develop climate-resilient varieties. Conclusively, under stress, rice has a bias towards codon usage which is correlated with GC content, gene expression level, and gene length.

## 1. Introduction

Rice is considered as a staple food for more than half of the world’s population due to its nutritional composition (a complete diet with carbohydrates, vitamins, fats, and minerals), and is consumed almost every day [1]. Like any other crop, rice is also exposed to several biotic/abiotic stresses, ultimately affecting its yield and quality and limiting its sustainable production [2]. To combat these different stresses, the rice plants re-program the expression of growth-, metabolism-, and resistance-related genes that either intensify or lower the concentration of several metabolites to protect against stresses [3]. This shift in the expression level of genes or proteins is correlated with several genomic features such as codon usage, gene length, amino acid/nucleotide composition, and rate of evolution [4]. Natural selection and mutational pressure also play a very important role in stress management by affecting the codon usage efficiency, giving rise to codon usage bias [5,6,7].

Codon usage bias (CUB) refers to the disparities in synonymous codons’ utilization frequency in a coding DNA sequence or gene. Organisms (plants or animals) prefer to use a particular set of synonymous codons during the translation process, and the selection of these synonymous codons is not random, but gives rise to biases in codon usage patterns, resulting in the altered expression level of a particular gene or protein [8,9]. The effects of codon usage were previously thought to be mainly mediated by its impacts on translation; however, studies have shown that codon usage strongly correlates with both protein and mRNA levels via gene codon optimization, resulting in the upregulation of protein and RNA levels [8,10]. Zhou et al. reported that the impact of codon usage on gene expression results mainly from effects on transcription and is largely independent of mRNA translation and mRNA stability. Further, the authors showed that codon usage can regulate mRNA transcription by influencing chromatin (histone H3 lysine 9 trimethylation) structure [10,11]. These results suggested that codon biases are an adaptation of protein coding sequences to both transcription and translation machineries. Thus, synonymous codons not only specify protein sequences and translation dynamics, but also help determine gene expression levels.

The CUB patterns vary among the species, genomes, or genes within the genome. Several studies have revealed the use of preferred codons in a particular pattern under a specific environmental/physiological condition that affects gene expression and contributes to the plant development process (including growth and defense) [12]. In addition, it is also reported that synonymous mutations that usually occur at the third codon position can switch some rare codons to frequently used codons, contributing to CUB [13]. It is also interesting to note that the t-RNA modification event (at wobble-base) allows or resists the specific codon–anticodon interaction producing the transcripts from biased codon patterns that after translation produce specific proteins [14].

The interactions between codon usage bias, gene expression, and underlying forces under stress conditions is a complex network that, if studied, can provide perspective to understand how genes respond in a specific (stress) condition. Thus, studying codon usage patterns under biotic and abiotic stress conditions is vital to understand evolution, adaptation, and plant diversity, and can be utilized further to design an optimized set of genes with specific codons. In the past, several studies have investigated the effect of codon usage bias in rice, Arabidopsis, and other plants [15,16,17,18]. However, how various environmental stresses, either biotic or abiotic, might influence codon usage, and these gene expression patterns in rice, have not been studied yet. In this context, it would be interesting to study the codon bias under different stress (abiotic or biotic) conditions in rice and its correlation with gene expression and nucleotide composition, following the mechanism through which the imposed stress condition influences the CUB pattern and contributes to the evolutionary process. Additionally, the possible target codons that could be used for editing the genome to develop resistance in rice has not been studied yet. Here, we aimed to study the codon usage bias pattern in rice using the rice transcriptome response data under various abiotic and biotic stresses, and investigated the correlation between codon usage bias, gene expression, and various codon usage indices, such as gene length, codon adaptation index (CAI), and relative synonymous codon usage (RSCU) in rice, and listed the possible targeted codons that could be genetically engineered for developing stress-resilient varieties in rice.

## 2. Results and Discussion

### 2.1. Differentially Expressed Genes Showed Similar Responses under Different Biotic or Abiotic Stress Conditions

Gene expression studies (transcriptome/RNA-Seq) were collected and reanalyzed for various stress conditions, i.e., drought, salt, temperature (high and low), bacterial leaf blight, blast, rice stripe, and dwarf rice disease in rice (Supplementary Table S1). It was noted that differentially expressed genes under different stress conditions shared some genes under each stress condition, i.e., abiotic or biotic stress. A total of 643 and 775 DEGs were shared among various abiotic and biotic stress conditions, respectively, and considered as major players contributing to stress resistance (Supplementary Table S2). Based on the expression pattern, the DEGs of each stress group (abiotic or biotic) were segregated as positively or negatively expressed genes and annotation was performed to explore their function details (Supplementary Tables S3 and S4). Further, the genes were categorized as high-, intermediate-, and low-expressed based on their expression profile and selected for downstream analysis. Because RNA-Seq data are usually considered a less-than-ideal source for single nucleotide variation (SNV) detection due to higher false positive rates for several reasons, such as higher complexity in alignment due to the RNA splicing, random errors introduced during reverse transcription, and PCR and RNA editing [19,20], it is not advisable to use the data for downstream analysis except in gene expression profiling. Studies have suggested that SNV detection difference between DNA-DNA pairs is only up to ~2%, while it approached ~20% when compared between DNA-RNA pairs. Considering that the discrepancies among SNVs detected by DNA and RNA through sequencing is quite high, the gene sequences were retrieved from the Nipponbare genome using the Rice Annotation Project (RAP) database (https://rapdb.dna.affrc.go.jp/ accessed on 22 January 2021) and used for downstream analysis.

### 2.2. Rice Genes Have a Bimodal Distribution of %GC Irrespective of Stress and Gene Expression Patterns

Previous studies have established the correlation between the %GC content and mutational pressure or natural selection as the dominating factors experienced, shaping the genome of a species [16,17,21,22]. Reports suggest that selection pressure acts on the first and second codon positions, while mutational pressure acts on the third codon position [7]. Hence, these forces play an important role in contributing to observed variation in the nucleotide composition of a species. To understand these forces and patterns, we analyzed the nucleotide composition of all the differentially expressed genes selected for biotic and abiotic stress conditions and subcategories (Table 1; Supplementary Tables S5 and S6). The gene names, accession numbers, and their functions are shown in Supplementary Tables S3 and S4.

Nucleotide composition analysis revealed that rice genes follow a bimodal distribution of %GC, where several genes had higher %GC while others had comparatively lower %GC (Supplementary Tables S5 and S6). Based on the nucleotide composition, the genes were grouped into two categories: high-GC genes (% GC content of more than 60%) and low-GC genes (% GC content of less than 60%), irrespective of their expression level and stress condition. This trend is expected in the rice genome and has been reported earlier [2,18]. Tatarinova et al. [23] also studied the cross-species GC3 pattern in animals as well as plants and noted that genes can be either GC-rich or GC-poor. Similarly, Wang and Hickey [18] also reported that the average %GC content of monocot or dicot plants was 57.8%, out of which 67.4% of genes fell under the category of high %GC, while 50.1% genes fell under the low %GC category. Our results are consistent with these studies, indicating that whether the genes are upregulated or downregulated does not affect the %GC distribution. The %GC content was highest at the third codon position, i.e., GC3, lowest at GC2, and intermediate at the GC1 position under both biotic or abiotic stress conditions (Supplementary Figures S1 and S2).

**Figure 1:**
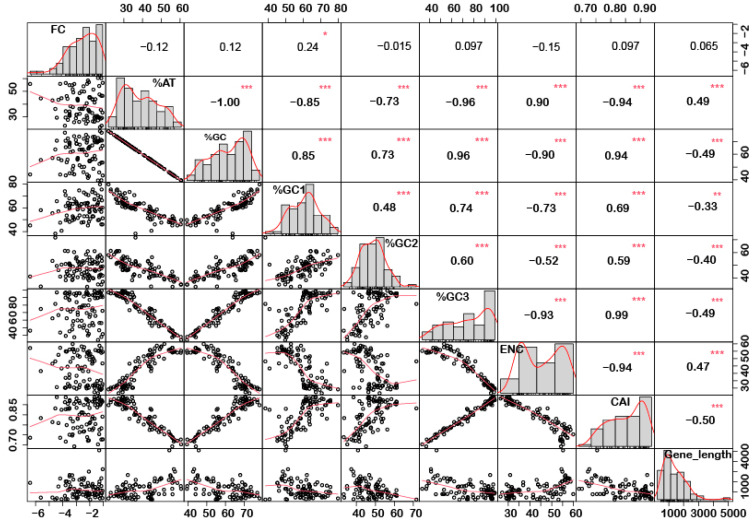
Correlation analysis among nucleotide composition, gene expression, gene length, ENC, and CAI of downregulated genes under abiotic stress. Based on the %GC, genes were grouped into two different categories, i.e., high and low %GC groups. %GC1 was positively correlated with gene expression, while %GC content (at the first, second and third codon positions) was negatively correlated with ENC and gene length while positively correlated with CAI. Asterisk indicate significant correlation, as *** with a *p*-value < 0.001, ** with *p*-value < 0.05 and * with a *p*-value < 0.01. FC = fold change; ENC = effective number of codons; CAI = codon adaptation index.

**Figure 2:**
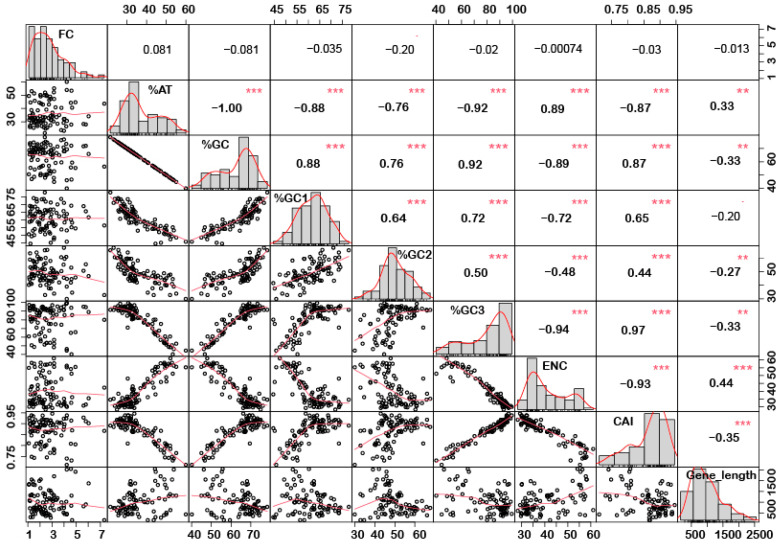
Correlation analysis among nucleotide composition, gene expression, gene length, ENC, and CAI of upregulated genes under abiotic stress. Based on the %GC, genes were grouped into two different categories, i.e., high and low %GC groups. %GC1 was positively correlated with gene expression, while %GC content (at the first, second and third codon positions) was negatively correlated with ENC and gene length while positively correlated with CAI. Asterisk indicate significant correlation, as *** with a *p*-value < 0.001, and ** with *p*-value < 0.05. FC = fold change; ENC = effective number of codons; CAI = codon adaptation index.

**Figure 3:**
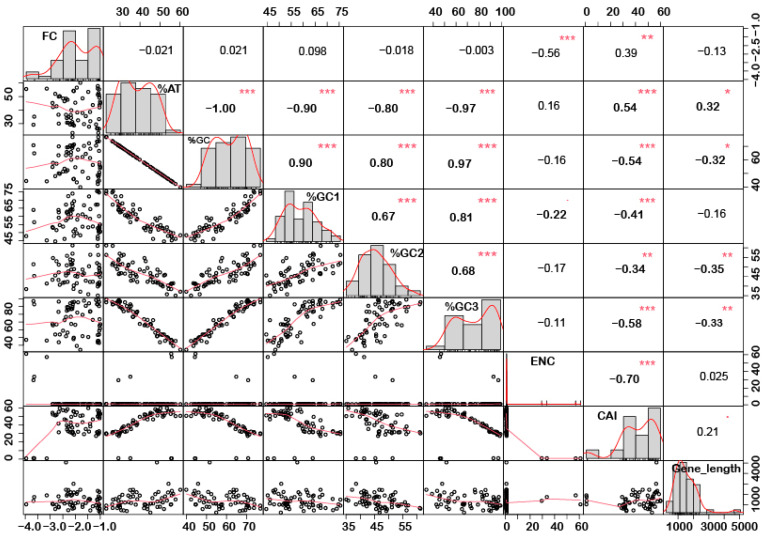
Correlation analysis among nucleotide composition, gene expression, gene length, ENC, and CAI of downregulated genes under biotic stress. A significant negative correlation was observed between gene expression and ENC, while %GC content (at the first, second and third codon positions) was negatively correlated with ENC and gene length while positively correlated with CAI. Asterisk indicate significant correlation, as *** with a *p*-value < 0.001, ** with *p*-value < 0.05 and * with a *p*-value < 0.01. FC = fold change; ENC = effective number of codons; CAI = codon adaptation index.

**Figure 4:**
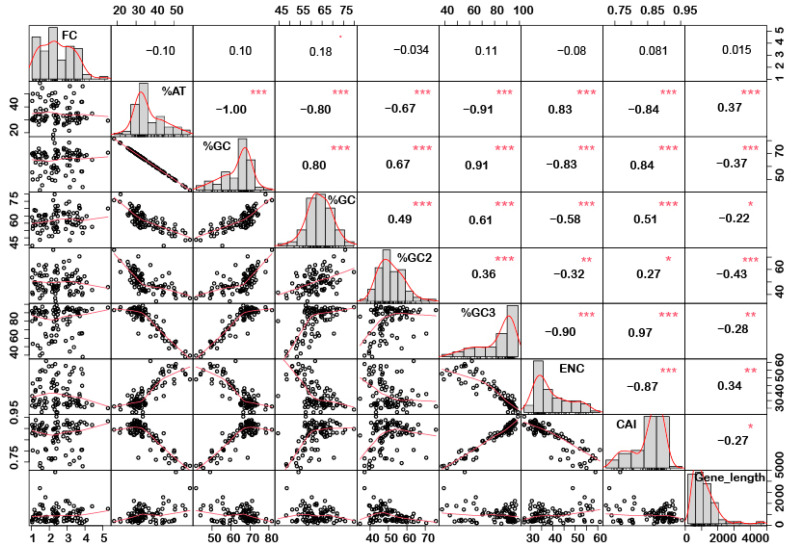
Correlation analysis among nucleotide composition, gene expression, gene length, ENC, and CAI of downregulated genes under biotic stress. Upregulated genes showed a positive correlation between %GC content (at the first, second, and third codon positions) and CAI, while a negative correlation was recorded for gene length and a positive correlation with CAI. Asterisk indicate significant correlation, as *** with a *p*-value < 0.001, ** with *p*-value < 0.05 and * with a *p*-value < 0.01. FC = fold change; ENC = effective number of codons; CAI = codon adaptation index.

In order to understand if there is any correlation between the nucleotide composition and gene expression (up or down) under stress conditions, correlation analysis was performed. Interestingly, it was noted that %GC1 had a positive correlation with gene expression in the case of downregulated genes under abiotic stress conditions (Figure 1), while no significant correlation was observed with upregulated genes (Figure 2). Additionally, there was no significant correlation recorded for the genes expressed (up- or downregulated) under biotic stress conditions (Figure 3 and Figure 4). Our results indicate that selection pressure which acts on the first or second codon position is correlated with the gene expression profile of negatively expressed genes under abiotic stress conditions.

### 2.3. Genes Length and %GC Content Are Directly Co-Related

Interestingly, a significant correlation was observed between the gene length and %GC or %AT content for most of the genes. The gene length was negatively correlated with the %GC content and positively correlated with the %AT content (Figure 1, Figure 2, Figure 3 and Figure 4). Based on %GC content and gene length, the genes were classified into three different clusters. Cluster 1 genes were shorter in length and had high GC% while longer genes had low %GC and were grouped as cluster 2. However, the third cluster had short-to-medium length and low GC content (Supplementary Figure S3). This pattern was witnessed among all the gene categories irrespective of the imposing stress factors and their expression level in the respective category. The average length of the low %GC genes was more than 2 kbp, while high %GC genes had an average length of around 1–1.5 kb. Although there is a wide range of individual gene lengths within each class, this difference suggests that the length of the rice genes was an important factor in the evolutionary increase in their GC content [18]. The propensity of raised GC content in rice lineage and inconsistencies in nucleotide composition have been reported to be linked with codon usage patterns [17]. Other monocots such as maize [14,15,24] and purple false brome [15], and dicots such as *Arabidopsis thaliana* [5] and *Mesona chinensis* [17], have shown similar effects on GC content, gene length, and CUB. It is evident from the previous studies that rapidly evolving genes are shorter, have more variable expression, and are GC3-rich in nature, while the evolutionarily stable genes tend to accumulate introns and increase the ORF length, and are larger [22,23]. Collectively, these results suggest that %GC content and gene length contribute positively during evolution.

### 2.4. Mutational Pressure Plays a Key Role in Shaping CUB in Downregulated Genes

**Figure 5:**
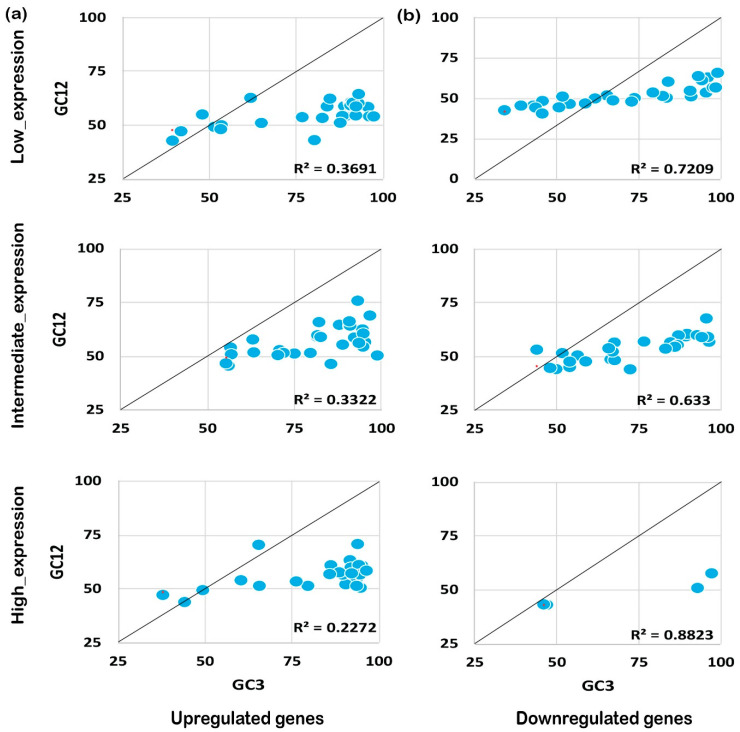
Neutrality plot analysis for genes expressed under biotic stress. The neutrality plot analysis for abiotic stress genes for upregulated (**a**) and downregulated (**b**) genes is shown. Neutrality plot analysis reveals the foremost influencing factor under biotic and abiotic stress by plotting GC12 against GC3. A regression coefficient close to 1 suggests that codon preference may be influenced by mutational pressure while a coefficient close to 0 suggests natural selection. Under biotic stress conditions, the upregulated genes are under higher mutational pressure than the genes expressed under abiotic stress conditions, while the downregulated genes seem to experience natural selection pressure but less than the genes expressed negatively under abiotic stress. As shown, the positively expressed genes are under mutation pressure while the negatively expressed genes are under natural selection pressure, irrespective of their expression level.

**Figure 6:**
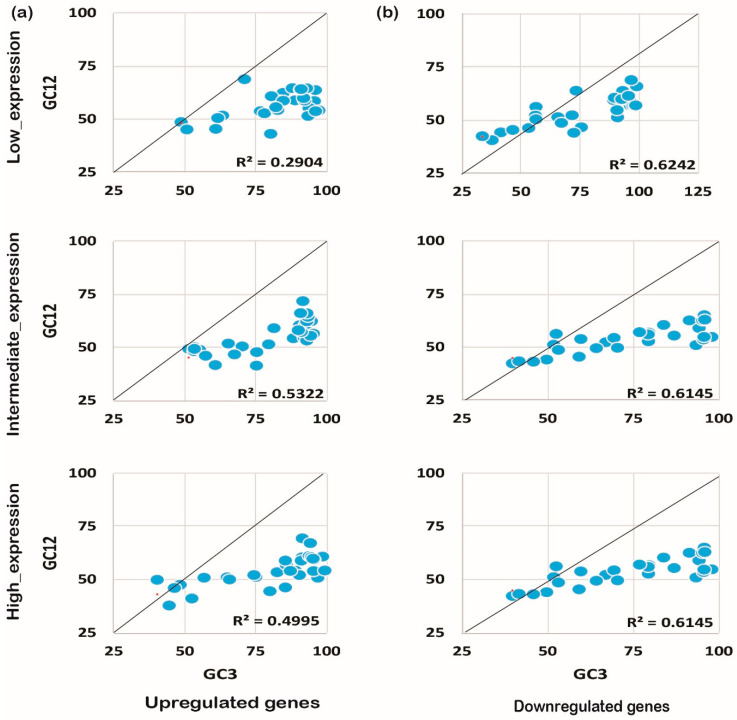
Neutrality plot analysis for genes expressed under abiotic stress. The expression pattern and the neutrality plot analysis for the genes expressed under biotic stress for upregulated (**a**) and downregulated (**b**) genes are evident.

The codon usage pattern is usually affected either by mutation pressure or the natural selection process, and eventually influences the expression pattern and plant growth/development processes [25]. To understand the foremost influencing factor under biotic and abiotic stress, we performed neutrality plot analysis. Neutrality plot analysis was performed by plotting GC12 against GC3. A regression coefficient close to 1 suggests that codon preference may be influenced by mutational pressure, while a coefficient close to 0 suggests natural selection [26]. Interestingly, we noticed that GC12 and GC3 had a significant correlation in the coding sequences expressed under biotic or abiotic stress conditions. It was also noted that upregulated genes under biotic stress conditions tended to be less influenced by mutational pressure (R^2^ ≤ 0.3, Figure 5a) rather than experiencing natural selection. On the contrary, downregulated genes showed higher effects from mutational pressure (R^2^ ≥ 0.5) for biotic stress and abiotic stress as shown in Figure 5b. A similar pattern was observed for the upregulated genes (R^2^ ≤ 0.5) expressed under abiotic stress conditions (Figure 6a,b). Collectively, the results indicate that downregulated genes have been experiencing mutational pressure in both biotic as well as abiotic stress conditions, while the upregulated genes were influenced by natural selection (Figure 5 and Figure 6). It is evident that mutational pressure or natural selection shapes the CUB in plants and other recently studied animals [27,28]; however, how up- or downregulated genes are influenced is not known. Our results summarize that, based on the gene expression pattern and nucleotide composition, different forces act on and reshape the CUB pattern and response in rice plants.

### 2.5. Codon Usage Biases Depends on the Gene Expression Profiles under Specific Stress Conditions

**Figure 7:**
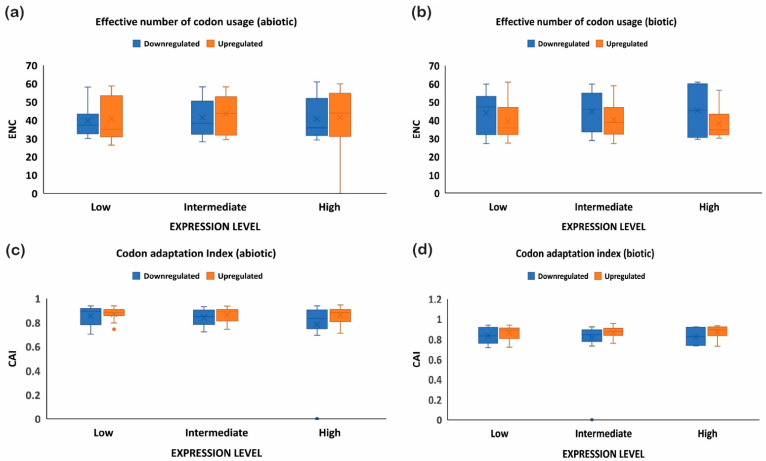
ENC and CAI analysis for upregulated and downregulated genes expressed under biotic and abiotic stress conditions. ENC analysis for (**a**) abiotic stress and (**b**) biotic stress, and CAI for (**c**) abiotic stress and (**d**) biotic stress. The upregulated genes have more ENC under abiotic stress than biotic stress, showing better adaptability, and vice versa. On the other hand, codon adaptation was higher in the upregulated genes than the downregulated genes.

The quantification ENC is used to understand the codon usage bias of a gene that is influenced by natural selection, mutation, or environmental factors [26]. It ranges between 20–61, where the value of 20 indicates that only one synonymous codon is used for each amino acid, while 61 indicates that all codons are used uniformly. Thus, the lower the value of ENC, the higher the bias [26,29]. Under abiotic stress conditions, the downregulated genes showed less codon usage bias compared to upregulated genes in all the subcategories, i.e., low-, intermediate-, and high-expressed genes (Figure 7a). On the contrary, under biotic stress conditions, downregulated genes had more biasness than the upregulated genes (Figure 7b). It was interesting to note that downregulated genes under biotic stress had more biasness than the abiotic stress condition, while upregulated genes were less biased, respectively. Intriguingly, it was observed that genes with a higher %GC had lower ENCs among all gene categories irrespective of their expression pattern and stress condition (Figure 1, Figure 2, Figure 3 and Figure 4). These results indicate that high %GC genes showed strong biases in codon usage as opposed to low %GC genes. To combat stress conditions, plants alter the expression of sets of genes that provide it resistance against the invading pathogen/stress condition and provide defense against the undesired conditions [30]. While gene expression can be upregulated or downregulated by imposed stress, the selection of variants under stress may have led to observed biases in codon usage. Our results agree with the previous study by Sharp et al. [31], who reported that the genes with high expression patterns have biases towards a particular set of codons. Likewise, a significant negative correlation was observed between ENC and gene expression level, indicating that highly expressed genes had low ENC and vice versa in our study (Figure 1, Figure 2, Figure 3 and Figure 4). Chamani et al. [2] also recorded a similar pattern of ENC and gene expression for the genes expressed under drought stress. Not only gene expression, but other factors, such as length of the gene, environmental stress, nucleotide composition, and t-RNA structure might also have influenced the ENC pattern and must be explored for further understanding.

### 2.6. Upregulated Genes Have Better Adaptation than Downregulated Genes

To further investigate the optimal codon usage pattern of rice genes under biotic and abiotic stress, the codon adaptation index was measured by taking the codon usage patterns of high-, intermediate-, and low-expressed genes (upregulated or downregulated). The range of CAI values is between 0 and 1; the higher the CAI values, the better adaptation towards the stressed condition [32]. The upregulated genes showed high CAIs (≥0.8 or above), while downregulated genes had lower CAI values (≤0.7) under abiotic stress, indicating the upregulated genes showed greater adaptation (Figure 7c). A similar trend was noticed under biotic stress conditions and also where downregulated genes showed reduced adaption compared to the upregulated genes (Figure 7d). Interestingly, it was observed that CAI was positively correlated with the gene expression profile of downregulated genes under biotic stress conditions, but negatively correlated with the %GC at the first, second, and third codon positions and ENC (Figure 1 and Figure 2). On the contrary, upregulated genes showed a positive correlation between CAI and %GC at the first, second, and third codon positions, and a negative correlation with CAI (Figure 3 and Figure 4). However, all the genes (upregulated or downregulated) showed a similar and positive correlation with the %GC and a negative correlation with ENC. All genes with high %GC and upregulated gene expression had a greater tendency to adapt towards the stress conditions than the downregulated or low %GC content genes. Collectively, these results suggest that under stress conditions, whether abiotic or biotic, genes that are upregulated experience more natural selection pressure and may increase their expression level by using a preferred codon pattern that might help the plant to resist the imposing stress condition. On the other hand, the downregulated genes experience more mutational pressure during stress conditions. Preferred codons are used in highly expressed genes, which help to accelerate the translation rate because of the high abundance of the decoding tRNAs [33]. Conversely, rare codons slow down protein translation and always accumulate in lowly expressed genes [33]. The upregulated genes could be used as target genes to increase plant defense because of their better adaptability against stress conditions, while the downregulated genes, based on their function, can be re-designed with an optimized set of codons for better adaptability and expression. Our results are supported by previous studies, where it was noticed that codon usage preference is associated with the stress received and plants, as a defense mechanism, optimize their codons, resulting in higher adaptability [27].

### 2.7. RSCU Analysis Revealed That Genes with GC at the Third Codon Position Have More Codon Bias

The expression of host genes is altered in response to biotic/abiotic stresses to combat invading pathogens or stress conditions [3]. This altered expression in the host (plant) provides resistance against the stressed condition and defends the plants [1]. The stressed conditions in plants have a direct correlation with expressed genes (either upregulated or downregulated) with codon usages derived due to either mutational pressure or natural selection [34]. To understand how the incoming stress (biotic/abiotic) is associated with the codon usage bias in rice, we compared the standard codon pattern of the rice with the genes expressed under stress (abiotic and biotic).

**Figure 8:**
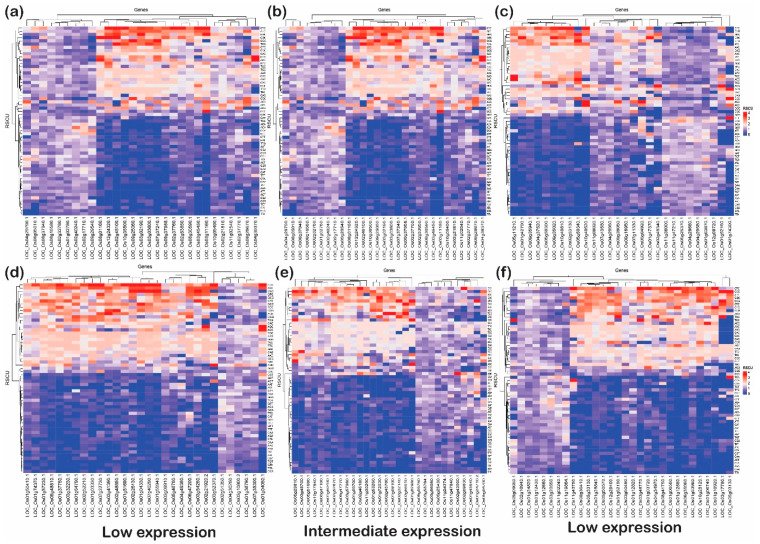
The heat plots of codon usage bias (CUB) under abiotic stress. The codon usage bias in the genes expressed under (**a**) low, (**b**) intermediate and (**c**) high gene expression categories for downregulated genes and CUB profile for (**d**) low-, (**e**) intermediate-, and (**f**) high-expressed genes for upregulated genes under abiotic stress condition in rice based on RSCU. The codons with AT at the third codon position are less biased than the codons with GC at the third nucleotide position.

**Figure 9:**
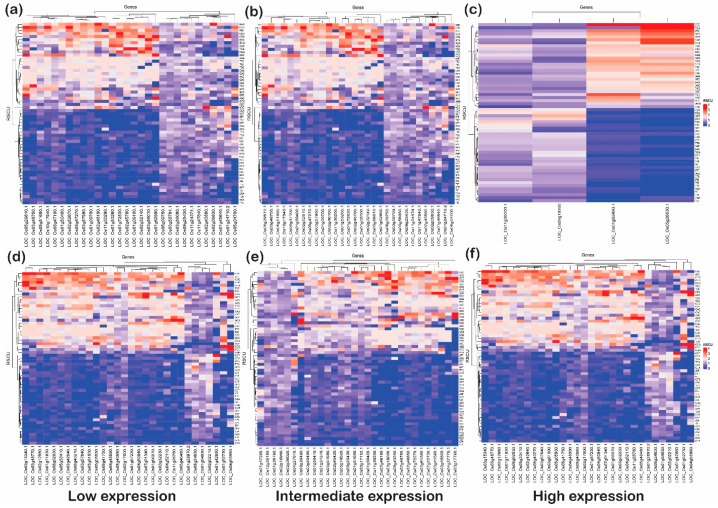
The heat plots of codon usage bias (CUB) under biotic stress. The codon usage bias in the genes expressed under (**a**) low, (**b**) intermediate and (**c**) high gene expression categories for downregulated genes and CUB profile for (**d**) low-, (**e**) intermediate- and (**f**) high-expressed genes for upregulated genes under abiotic stress condition in rice based on RSCU. The codons with AT at the third codon position are less biased than the codons with GC at the third nucleotide position.

This analysis articulated the change in codon usage patterns of a specific amino acid. The heat plot was generated for each category (low, intermediate, and high) for upregulated and downregulated genes under biotic as well as abiotic stress (Figure 8 and Figure 9). These plots indicate that genes with G or C end codons at the third position or high %GC had more RSCU, and were highly preferred to those with A or T at the third codon position. On the other hand, codons with A/T at the third nucleotide position had lower RSCU and expression. It was observed that TAC (tyrosine), CAC (histidine), AAC (asparagine), GAC (aspartate), and TGC (cysteine) were moderately preferred in low- and high-expressed genes, but highly preferred in intermediately expressed genes under abiotic stress conditions. Likewise, CTA and TTA (leucine) and ATA (isoleucine) were not at all preferred in low- and high-expressed genes but somewhat preferred for intermediately expressed genes under abiotic stress. Interestingly, TTC (phenylalanine), ATC (isoleucine) and CTC (leucine) were preferred in all subcategories of upregulated as well as downregulated genes under abiotic stress. However, in biotic stress conditions, TTC (phenylalanine) was favored in all sub-categories of downregulated genes but less preferred in upregulated genes. On the other hand, ATC (isoleucine) and CTC (leucine) were highly preferred in upregulated as well as downregulated genes of all the sub-categories under biotic stress. In addition, ACC (threonine), GCC (alanine), TAC (tyrosine), CAC (histidine), AAC (asparagine), GAC (aspartate) and TGC (cysteine), with C at the third codon position, were more preferred than any with an A or T nucleotide at the third codon position. However, all these codons were highly preferred in the downregulated genes when compared with upregulated genes. Additionally, GGC (glycine), CCC (proline), and TTG (leucine) were the codons that were preferred over the A/T codons at the third codon positions in any categories (low, intermediate, and high) or stress (biotic or abiotic) condition of upregulated and downregulated genes. The widely used codons for serine and alanine in rice, GCC and TCC, were not preferred in highly expressed genes under biotic or abiotic stress, but GCG and TCG were highly preferred. Similarly, GAC and CCG were preferred for aspartic acid and proline, respectively, despite the standard codons GAT and CCA in rice. This trend of gene expression, RSCU and codon preferences has been observed in rice, Arabidopsis, and prokaryote species. For example, Barozi and Din [5] indicated that 43% of codons showed different CUB under cold stress-resistant genes in *A. thaliana*, while 63% of codons were observed with different CUB analyses of cold stress-resistant genes in rice. The results from their study suggested that plants use codon optimization programs for handling stress. Our results are consistent with these studies, indicating that irrespective of the incoming stress condition, the rice plant prefers to have optimized codons to tackle stress conditions. Hypothetically, the plant favors specific codons to combat the stressed condition. Based on this analysis, the favorable codons under stress were identified, which can be further used to design the optimized set of genes for the development of stress-tolerant rice varieties (Table 2). Studies in the past have shown the potential of codon optimization to achieve the enhanced expression of heterologous genes to attain the desired feature in the organisms. Here, it is important to note that rice plants prefer to have C or G at wobble positions rather than A or T. These preferences at wobble positions may alter mRNA recognition via RNA decay pathways; however, it is a hypothesis that needs to be tested to be proven correct. However, it was noted that there were some genes that utilized AT or GC codons (third position) uniformly. These genes, based on their function, can be used for better adaptability against the stress received (Table 3).

Further, genes showing maximum codon bias were analyzed using Blast2GO tools to annotate their function (molecular, biological, or cellular). These genes were found to mediate either transcriptional activities, enzymatic activities, protein binding or other metabolic/cellular/molecular activities (Supplementary Table S7). Interestingly, the functional domain analysis using InterPro evidenced that most of the genes were either an inhibitor or transcription factor, or an enzyme such as WRKY, MYB, GATA, etc., which play important roles in plant growth development and defense.

## 3. Material and Methods

### 3.1. Differential Gene Expression in Rice under Abiotic and Biotic Stress Conditions

A systemic literature survey was performed to identify rice transcriptome studies under various abiotic or biotic stress conditions, i.e., drought, salt, temperature (high and low), bacterial leaf blight, blast, rice stripe, and dwarf rice disease in rice. The gene expression data from the transcriptome studies which had at least two replicates, untreated control vs. treatment samples, and containing aboveground tissue conducted with wildtype plants only were chosen, as discussed by Cohen and Leach [6], and utilized for this study. Further, the genomic sequence of DEGs were retrieved from the Nipponbare reference genome (v7), but not from the SRA library, as discussed above. The functional annotation of the genes, their physical position, and the functional domains were retrieved using the Rice Annotation Project (RAP) database (https://rapdb.dna.affrc.go.jp/ accessed on 22 January 2021) tools [35,36]. The genes were first sorted as upregulated and downregulated from both groups, i.e., biotic and abiotic stress, and then further categorized as (A) highly upregulated/downregulated (≥3-fold or more), (B) intermediately upregulated/downregulated (≥2-fold) or (C) low upregulated/downregulated (≥1-fold). While choosing the genes for downstream processing, preference was given to the already characterized genes whose function was well known. A literature survey revealed that many of these genes have been annotated and have shown resistance against blast, blight pathogens, and drought and salinity stresses, and fall into different functional categories such as transcription factors, proteins, dehydrogenase membrane-bound proteins, etc. Gene Ontology and enrichment analysis of the genes showing maximum codon bias was conducted using the Blast2GO software (version 3.0) with default parameters [37].

### 3.2. Nucleotide Composition of Selected Genes

The nucleotide composition, i.e., AT and GC content of all the selected genes at the 1st, 2nd and 3rd nucleotide positions (AT1, AT2, AT3 and GC1, GC2, GC3, respectively), were calculated using CAIcal as discussed by Wang et al. [38,39].

### 3.3. Neutrality Plot Analysis

Mutation pressure and natural selection are two important factors that influence codon preference [40]. To investigate the effect of mutational and natural selection on codon usage, neutrality plot analysis was performed to determine the main factor influencing codon usage bias by plotting GC12 against GC3 with a scatterplot. A slope near 0 indicates high influence by natural selection, whereas a slope close to 1 describes biases due to mutational pressure and a slope of 0.5 means complete neutrality [40]. The values for GC12 were calculated by the mean of GC1 and GC2, and it was calculated using CAIcal [38,39]. GC3 and GC12 of each coding sequence (CDS) were plotted to analyze the correlation between the base compositions of three different sites of codons and to determine the main influencing factors of codon bias.

### 3.4. Relative Synonymous Codon Usage (RSCU) Analysis

RSCU was calculated as the ratio between observed and expected codon frequencies with the supposition that all the codons for a particular amino acid are used equally. The RSCU of all selected genes associated with biotic and abiotic stress in rice were calculated using CAIcal [38] and ACUA [41] programs, excluding the start (ATG) and stop codons (TAG, TAA, TGA), as well as the Trp amino acid, which is encoded by a single codon. The codon usage bias was calculated using the MEGA X program [42] to determine the codon usage patterns under biotic and abiotic stress and compare them with standard codon usage patterns in rice.

### 3.5. Effective Number of Codon Usage (ENC) Analysis

The effective number of codon usage (ENC) refers to the number of unique codons found in a gene. This value can be in a range between 20 (where there is an extreme bias towards using only one codon for each amino acid) and 61 (representing the use of all synonymous codons). If there are fewer than 60 synonymous codons used in a particular gene, then CUB may be present (since Trp has only one codon). ENC was calculated as discussed by Wang et al. using CAIcal [38] and CodonW program.

### 3.6. Codon Adaptation Index (CAI) Analysis

CAI is another widely used method for the evaluation of CUB. It measures the similarity between the frequency of synonymous codons used by a gene and a reference set. The range of the CAI value is between 0 and 1. The rice codon usage from the Codon Usage Database (https://www.kazusa.or.jp/codon/ accessed on 30 January 2021) was used as a reference set [43]. The CAI was calculated as discussed by Gao et al. [44].

## 4. Conclusions

Codon usage bias plays an important role in shaping genomes and genes in unicellular and multicellular species. In the current study, it was observed that downregulated genes experience more mutational pressure, while upregulated genes are under natural selection pressure. Stress plays an important role in shaping the codon usage pattern in plants, and genes with high %GC or with G or C at the third codon position showed more bias towards specific amino acids. Collectively, the results indicate that downregulated genes experience mutational pressure in both abiotic as well as biotic stress conditions, while the upregulated genes experience natural selection. Based on the results of this study, we concluded that natural selection is the dominating pressure driving the codon usage bias in upregulated genes, indicating the effect of stress conditions and the expression of genes. Thus, our findings will allow readers to understand the forces behind the correlation between the gene expression pattern under different stress conditions and codon usage bias in rice.

## Figures and Tables

**Table 1:** Average %GC, %GC1, %GC2, %GC, CAI, and ENC of upregulated and downregulated genes under abiotic and biotic stress conditions.

Abiotic Stress							
Downregulated Genes							
Gene Categories	%AT	%GC	%GC1	%GC2	%GC3	CAI	ENC
Low-expressed (≤1)	37.84	62.15	61.52	47.84	77.10	0.854	40.91
Intermediate-expressed (≤2)	39.49	60.50	59.83	47.67	73.99	0.842	43.49
High-expressed (≤3)	40.70	59.29	56.90	48.25	72.74	0.83	44.47
Average of low-, intermediate-, and high- expressed genes	39.31	60.68	59.48	47.91	74.65	0.84	42.92
**Upregulated Genes**							
Low-expressed (≤1)	34.84	65.15	61.64	51.78	82.03	0.87	39.65
Intermediate-expressed (≤2)	37.20	62.79	60.92	48.45	79.00	0.86	41.39
High-expressed (≤3)	37.52	62.47	60.17	47.52	79.71	0.861	40.60
Average of low-, intermediate-, and high-expressed genes	36.52	63.47	60.91	49.25	80.25	0.865	40.55
**Biotic Stress**							
**Downregulated Genes**							
Low-expressed (≤1)	41.63	58.36	58.16	45.73	71.19	0.83	43.94
Intermediate-expressed (≤2)	39.38	60.61	59.53	48.30	74.01	0.84	44.70
High-expressed (≤3)	43.92	56.07	53.33	44.15	70.73	0.82	45.32
Average of low-, intermediate-, and high-expressed genes	40.74	59.25	61.95	50.34	81.09	0.83	44.38
**Upregulated Genes**							
Low-expressed (≤1)	36.72	63.27	59.90	50.62	79.28	0.86	39.68
Intermediate-expressed (≤2)	34.91	65.08	63.37	50.74	81.12	0.86	40.33
High-expressed (≤3)	34.96	65.03	62.58	49.65	82.85	0.87	38.25
Average of low-, intermediate-, and high-expressed genes	35.53	64.46	58.48	46.82	72.46	0.86	39.42

**Table 2:** Catalog of potentially beneficial replacement targets for synonymous change in rice genes.

S. No.	Amino Acid	Codons	Favorable Synonymous Codon under Stress (Biotic, Abiotic)
1	Phe	TTT	TTC
		TTC	
2	Leu	TTA	CTC, CTG
		TTG	
		CTT	
		CTC	
		CTA	
		CTG	
3	Ile	ATT	ATC
		ATC	
		ATA	
4	Met	ATG	ATG
5	Val	GTT	GTC, GTG
		GTC	
		GTA	
		GTG	
6	Ser	TCT	TCC, TCG, AGC
		TCC	
		TCA	
		TCG	
		AGT	
		AGC	
7	Pro	CCT	CCC, CCG
		CCC	
		CCA	
		CCG	
8	Thr	ACT	ACC, ACG
		ACC	
		ACA	
		ACG	
9	Ala	GCT	GCC, GCG
		GCC	
		GCA	
		GCG	
10	Tyr	TAT	TAC
		TAC	
		TAA	
		TAG	
11	His	CAT	CAC
		CAC	
12	Gln	CAA	CAG
		CAG	
13	Asn	AAT	AAC
		AAC	
14	Lys	AAA	AAG
		AAG	
15	Asp	GAT	GAC
		GAC	
16	Glu	GAA	GAG
		GAG	
17	Cys	TGT	TGC
		TGC	
18	Arg	CGT	CGC, CGG, AGG
		CGC	
		CGA	
		CGG	
		AGA	
		AGG	
19	Gly	GGT	GGC, GGG
		GGC	
		GGA	
		GGG	
20	Trp	TGG	TGG

**Table 3:** Genes expressed under biotic and abiotic stress conditions with uniform usage of codons.

Locus	Function	Locus	Function
LOC_Os01g02780	ARK protein	LOC_Os01g12430	Response to drought and chilling in tolerant genotypes
LOC_Os02g17710	Leucine-rich repeat domain containing protein	LOC_Os01g24820	Disease resistance protein domain containing protein
LOC_Os02g32780	Pentatricopeptide repeat domain containing protein	LOC_Os03g20550	WRKY transcription factor, auxin response
LOC_Os02g40240	Protein kinase, core domain containing protein	LOC_Os03g58764	F-box domain containing protein
LOC_Os08g16580	Leucine-rich repeat, N-terminal domain containing protein	LOC_Os01g42860	Salt and osmotic stress tolerance
LOC_Os08g37940	Haloacid dehalogenase-like hydrolase domain containing protein	LOC_Os01g52730	S40 gene family gene
LOC_Os01g24710	Resistance to M. oryzae	LOC_Os01g54030	Cytosolic NADP malic enzyme
LOC_Os01g45640	Twin-arginine translocation pathway protein	LOC_Os02g16940	Subtilise gene
LOC_Os02g09830	Drought resistance	LOC_Os02g36020	Ent-kaurene synthase (KS)-like diterpene synthase
LOC_Os02g43560	WRKY-like DNA-binding protein	LOC_Os04g55980	Conserved hypothetical protein
LOC_Os03g56060	CSLC9	LOC_Os11g24374	Serine carboxypeptidase
LOC_Os05g04700	Cold stress response	LOC_Os11g36000	Leucine-rich repeat-containing containing protein

## Data Availability

The gene expression datasets used in this study were generated by Cohen and Leach 2019 from the SRA data available in the NCBI database under following accession numbers SRP071248, SRP052306, SRP113286, SRP101342, SRP004651, SRP056884, SRP049040, SRP076382, SRP049444, SRP065503, and SRP115030. Further downstream processed data files can be received from the corresponding author on reasonable request.

## Supplementary Materials

The following supporting information can be downloaded at: https://www.mdpi.com/article/10.3390/ijms24021098/s1. The supporting information is provided as Excel files.

## References

[1] Vasanthi S., Dass J.F.P. (2018). Comparative genome-wide analysis of codon usage of different bacterial species infecting Oryza sativa. J. Cell. Biochem..

[2] Chamani Mohasses F., Solouki M., Ghareyazie B., Fahmideh L., Mohsenpour M. (2020). Correlation between gene expression levels under drought stress and synonymous codon usage in rice plant by in-silico study. PLoS ONE.

[3] Tyagi S., Mulla S.I., Lee K.J., Chae J.C., Shukla P. (2018). VOCs-mediated hormonal signaling and crosstalk with plant growth promoting microbes. Crit. Rev. Biotechnol..

[4] Gout J.F., Kahn D., Duret L., Consortiu P.P.-G. (2010). The Relationship among Gene Expression, the Evolution of Gene Dosage, and the Rate of Protein Evolution. PLoS Genet..

[5] Barozai M.Y.K., Din M. (2014). The Relationship between Codon Usage Bias and Cold Resistant Genes. Pak. J. Bot..

[6] Cohen S.P., Leach J.E. (2019). Abiotic and biotic stresses induce a core transcriptome response in rice. Sci. Rep..

[7] Comeron J.M. (2004). Selective and mutational patterns associated with gene expression in humans: Influences on synonymous composition and intron presence. Genetics.

[8] Lopez J.L., Lozano M.J., Lagares A., Fabre M.L., Draghi W.O., Del Papa M.F., Pistorio M., Becker A., Wibberg D., Schluter A. (2019). Codon Usage Heterogeneity in the Multipartite Prokaryote Genome: Selection-Based Coding Bias Associated with Gene Location, Expression Level, and Ancestry. Mbio.

[9] Oldfield C.J., Peng Z.L., Uversky V.N., Kurgan L. (2020). Codon selection reduces GC content bias in nucleic acids encoding for intrinsically disordered proteins. Cell. Mol. Life Sci..

[10] Zhou Z., Dang Y., Zhou M., Li L., Yu C.H., Fu J., Chen S., Liu Y. (2016). Codon usage is an important determinant of gene expression levels largely through its effects on transcription. Proc. Natl. Acad. Sci. USA.

[11] Zhou Z., Dang Y., Zhou M., Yuan H., Liu Y. (2018). Codon usage biases co-evolve with transcription termination machinery to suppress premature cleavage and polyadenylation. eLife.

[12] Qin F., Shinozaki K., Yamaguchi-Shinozaki K. (2011). Achievements and Challenges in Understanding Plant Abiotic Stress Responses and Tolerance. Plant Cell Physiol..

[13] Mitra S., Ray S., Banerjee R. (2016). Synonymous codons influencing gene expression in organisms. Res. Rep. Biochem..

[14] Liu Y. (2020). A code within the genetic code: Codon usage regulates co-translational protein folding. Cell Commun. Signal.

[15] Liu H., He R., Zhang H., Huang Y., Tian M., Zhang J. (2010). Analysis of synonymous codon usage in Zea mays. Mol. Biol. Rep..

[16] Sahoo S., Das S.S., Rakshit R. (2019). Codon usage pattern and predicted gene expression in Arabidopsis thaliana. Gene.

[17] Tang D., Wei F., Cai Z., Wei Y., Khan A., Miao J., Wei K. (2021). Analysis of codon usage bias and evolution in the chloroplast genome of Mesona chinensis Benth. Dev. Genes Evol..

[18] Wang H.C., Hickey D.A. (2007). Rapid divergence of codon usage patterns within the rice genome. BMC Evol. Biol..

[19] Guo Y., Zhao S.L., Sheng Q.H., Samuels D.C., Shyr Y. (2017). The discrepancy among single nucleotide variants detected by DNA and RNA high throughput sequencing data. BMC Genom..

[20] Li M.Y., Wang I.X., Li Y., Bruzel A., Richards A.L., Toung J.M., Cheung V.G. (2011). Widespread RNA and DNA Sequence Differences in the Human Transcriptome. Science.

[21] Song H., Liu J., Song Q., Zhang Q., Tian P., Nan Z. (2017). Comprehensive Analysis of Codon Usage Bias in Seven Epichloë Species and Their Peramine-Coding Genes. Front. Microbiol..

[22] Tatarinova T., Elhaik E., Pellegrini M. (2013). Cross-species analysis of genic GC3 content and DNA methylation patterns. Genome Biol. Evol..

[23] Tatarinova T.V., Alexandrov N.N., Bouck J.B., Feldmann K.A. (2010). GC3 biology in corn, rice, sorghum and other grasses. BMC Genom..

[24] Chu D., Wei L. (2019). Parsing the synonymous mutations in the maize genome: Isoaccepting mutations are more advantageous in regions with codon co-occurrence bias. BMC Plant Biol..

[25] Mazumder G.A., Uddin A., Chakraborty S. (2020). Analysis of codon usage pattern of mitochondrial ND genes in Platyhelminthes. Mol. Biochem. Parasit..

[26] Fuglsang A. (2008). Impact of bias discrepancy and amino acid usage on estimates of the effective number of codons used in a gene, and a test for selection on codon usage. Gene.

[27] Biswas K.K., Palchoudhury S., Chakraborty P., Bhattacharyya U.K., Ghosh D.K., Debnath P., Ramadugu C., Keremane M.L., Khetarpal R.K., Lee R.F. (2019). Codon Usage Bias Analysis of Citrus tristeza virus: Higher Codon Adaptation to Citrus reticulata Host. Viruses.

[28] Muthabathula P., Suneetha S., Grace J.R. (2018). Genome-wide codon usage bias analysis in Beauveria bassiana. Bioinformation.

[29] Wright F. (1990). The Effective Number of Codons Used in a Gene. Gene.

[30] Liberatore K.L., Dukowic-Schulze S., Miller M.E., Chen C.B., Kianian S.F. (2016). The role of mitochondria in plant development and stress tolerance. Free Radic. Biol. Med..

[31] Sharp P.M., Li W.H. (1987). The Codon Adaptation Index—A Measure of Directional Synonymous Codon Usage Bias, and Its Potential Applications. Nucleic Acids Res..

[32] Nath Choudhury M., Uddin A., Chakraborty S. (2017). Codon usage bias and its influencing factors for Y-linked genes in human. Comput. Biol. Chem..

[33] Xu Y.C., Liu K.S., Han Y., Xing Y.Z., Zhang Y.X., Yang Q.Y., Zhou M. (2021). Codon usage bias regulates gene expression and protein conformation in yeast expression system P. pastoris. Microb. Cell Fact..

[34] Williford A., Demuth J.P. (2012). Gene expression levels are correlated with synonymous codon usage, amino acid composition, and gene architecture in the red flour beetle, Tribolium castaneum. Mol. Biol. Evol..

[35] Kawahara Y., de la Bastide M., Hamilton J.P., Kanamori H., McCombie W.R., Ouyang S., Schwartz D.C., Tanaka T., Wu J.Z., Zhou S.G. (2013). Improvement of the Oryza sativa Nipponbare reference genome using next generation sequence and optical map data. Rice.

[36] Sakai H., Lee S.S., Tanaka T., Numa H., Kim J., Kawahara Y., Wakimoto H., Yang C., Iwamoto M., Abe T. (2013). Rice Annotation Project Database (RAP-DB): An Integrative and Interactive Database for Rice Genomics. Plant Cell Physiol..

[37] Conesa A., Gotz S., Garcia-Gomez J.M., Terol J., Talon M., Robles M. (2005). Blast2GO: A universal tool for annotation, visualization and analysis in functional genomics research. Bioinformatics.

[38] Puigbo P., Bravo I.G., Garcia-Vallve S. (2008). CAIcal: A combined set of tools to assess codon usage adaptation. Biol. Direct..

[39] Wang Q.Q., Lyu X.L., Cheng J.S., Fu Y.P., Lin Y., Abdoulaye A.H., Jiang D.H., Xie J.T. (2022). Codon Usage Provides Insights into the Adaptive Evolution of Mycoviruses in Their Associated Fungi Host. Int. J. Mol. Sci..

[40] Chakraborty S., Yengkhom S., Uddin A. (2020). Analysis of codon usage bias of chloroplast genes in Oryza species: Codon usage of chloroplast genes in Oryza species. Planta.

[41] Vetrivel U., Arunkumar V., Dorairaj S. (2007). ACUA: A software tool for automated codon usage analysis. Bioinformation.

[42] Kumar S., Stecher G., Li M., Knyaz C., Tamura K. (2018). MEGA X: Molecular Evolutionary Genetics Analysis across Computing Platforms. Mol. Biol. Evol..

[43] Nakamura Y., Gojobori T., Ikemura T. (2000). Codon usage tabulated from international DNA sequence databases: Status for the year 2000. Nucleic Acids Res..

[44] Gao Y., Lu Y., Song Y., Jing L. (2022). Analysis of codon usage bias of WRKY transcription factors in Helianthus annuus. BMC Genom. Data.

