# Supplementary material for: Codon Usage Provide Insights into the Adaptation of Rice Genes under Stress Condition
Source: Int J Mol Sci. 2023 Jan 6;24(2):1098. doi: 10.3390/ijms24021098 (PMC9861248; doi:10.3390/ijms24021098)
Supplement: Supplementary file 1 [file ijms-24-01098-s001.zip › Supplementary_information_CUB.pdf]

Codon usage bias and gene expression level in rice under abiotic stress conditions

Swati Tyagi[1], Pramod Gorakhanath Kabade[1], Niranjani Gnanapragasam[2], Uma Maheshwar Singh[1], Anoop Kishor Singh Gurjar[1], Ashutosh Rai[1,3], Pallavi Sinha[2], Arvind Kumar[1, 4], Vikas Kumar Singh[1, 2,*]

[1]International Rice Research Institute, South Asia Regional Centre (ISARC), Varanasi, India

[2]International Rice Research Institute (IRRI), South-Asia Hub, ICRISAT Campus, Hyderabad, India

[3]Banda University of Agriculture and Technology, Banda

[4]International Crops Research Institute for the Semi-Arid Tropics, Hyderabad, India

***Correspondence:** v.k.singh@irri.org

Supplementary information

Supplementary figures

Fig. S1. Nucleotide composition of rice genes expressed under abiotic stress. (a-c) Upregulated genes (d-f) downregulated genes with low, intermediate, and high expression level respectively.

Fig. S2. Nucleotide composition of rice genes expressed under biotic stress. (a-c) Upregulated genes (d-f) downregulated genes with low, intermediate, and high expression level respectively.

Fig. S3. The Correlation analysis between %GC and gene length. The gene length and %GC is directly correlated, and the genes are distributed into three different clusters. (a) abiotic stress (b) biotic stress.

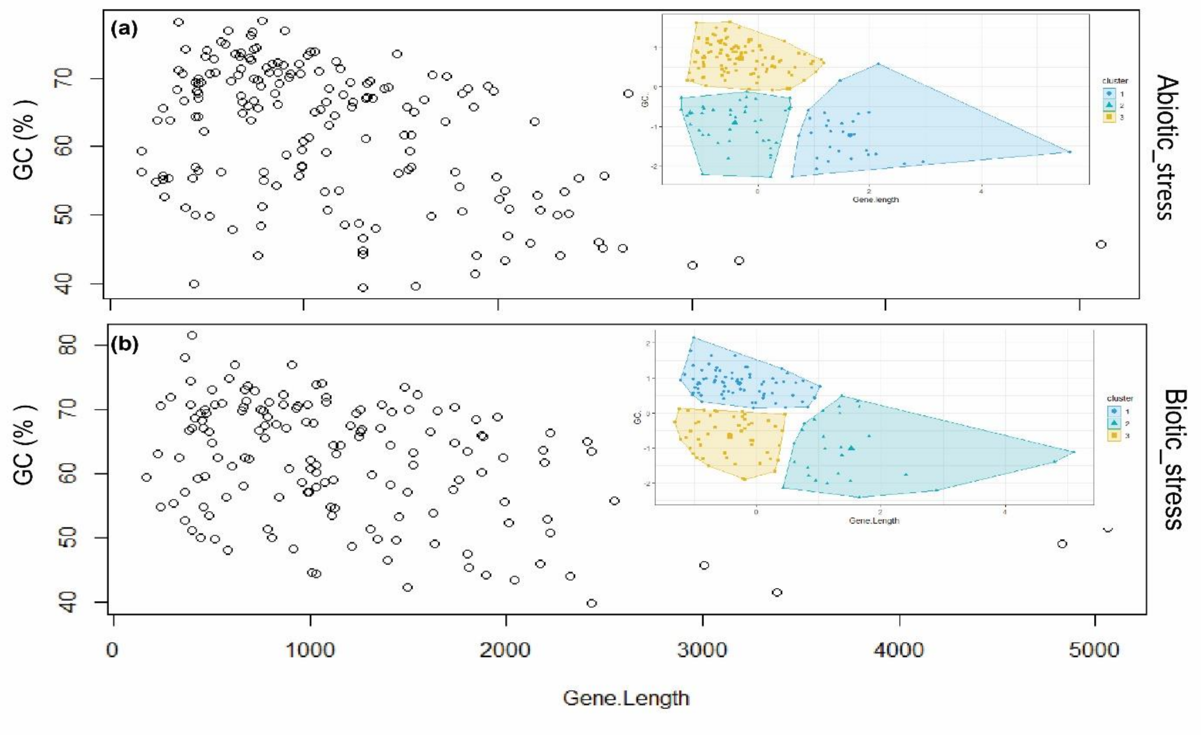

Supplementary Table (supplied as Excel files)

Supplementary Table S1 List of SRA libraries, their accession and cultivars used in current study

Supplementary Table S2. Differentially expressed genes in rice under abiotic and biotic stress conditions

Supplementary Table S3. Sorting, function predication of differentially expressed genes under abiotic stress condition.

Supplementary Table S4. Sorting, function predication of differentially expressed genes under biotic stress condition.

Supplementary Table S5: Nucleotide composition, CAI and ENC in the genes expressed under abiotic stress conditions

Supplementary Table S6: Nucleotide composition, CAI and ENC in the genes expressed under biotic stress conditions
